# Reconstruction with omental flap and negative pressure wound therapy after total pelvic exenteration of anal fistula cancer: a case report

**DOI:** 10.1186/s40792-022-01472-z

**Published:** 2022-06-20

**Authors:** Akira Komono, Gumpei Yoshimatsu, Ryuji Kajitani, Yoshiko Matsumoto, Naoya Aisu, Suguru Hasegawa

**Affiliations:** 1 Department of Gastroenterological Surgery, Fukuoka University Hospital, 7-45-1 Nanakuma, Jonan-ku, Fukuoka, 814-0180 Japan; 2 Department of Regenerative Medicine and Transplantation, Fukuoka University Hospital, 7-45-1 Nanakuma, Jonan-ku, Fukuoka, 814-0180 Japan

**Keywords:** Anal fistula cancer, Omental flap, Negative pressure wound therapy, Total pelvic exenteration

## Abstract

**Introduction:**

Surgery for anal fistula cancer (AFC) associated with Crohn’s disease usually entails extensive perineal wounds and dead space in the pelvis, which is often filled with a myocutaneous flap. However, use of a myocutaneous flap is invasive. We report a case of total pelvic exenteration (TPE) for AFC in which a myocutaneous flap was avoided by using an omental flap and negative pressure wound therapy (NPWT).

**Case presentation:**

The patient was a 47-year-old woman who had been treated for Crohn’s disease involving the small and large intestine for 30 years and had repeatedly developed anal fistulas. She was referred with a diagnosis of AFC that had spread extensively in the pelvis. We performed laparoscopic TPE via a transperineal endoscopic approach. To prevent infection in the large skin defect and extensive pelvic dead space postoperatively, the perineal wound was reconstructed using an omental flap and NPWT. During 20 days of NPWT, the wound steadily decreased in size and closed on postoperative day (POD) 20. She was discharged without complications on POD 30.

**Discussion:**

NPWT is useful for preventing perineal wound infection and promoting granulation tissue formation. However, direct contact with the intestine may lead to intestinal perforation. In this case, the combination of an omental flap with NPWT effectively prevented surgical site infection. The flap filled the large pelvic dead space and physically separated the intestine from the polyurethane foam used for NPWT.

**Conclusion:**

NPWT and an omental flap may become an option when performing TPE.

## Background

Unlike rectal and anal canal cancers, anal fistula cancer requires extensive perineal skin incision and wide perineal tissue excision because of the likelihood of positive margins and local recurrence [1, 2]. Infection of the pelvic cavity and perineal wound is also common because of the large dead space created in the pelvis during surgery and the potential for bacterial contamination of the surgical field around the perineum. Prevention of perineal infection is important because such infection contributes to poor quality of life and affects survival [3]. A myocutaneous flap using a gracilis muscle, the gluteus maximus, or rectus abdominis is often used to prevent infection-related complications [4, 5]. However, myocutaneous flaps are associated with problems such as reduced ability to perform activities of daily living due to impairment of gait, muscle weakness, postoperative infection, and necrosis of the flap.

There have been no reports of a minimally invasive method for filling the dead space that avoids using a myocutaneous flap in TPE with an extensive skin defect. There are, however, some reports of abdominoperineal resection (APR) using an omental flap to reduce postoperative perineal wound infection [6, 7]. There are also reports of incisional negative pressure wound therapy (NPWT) and open NPWT having prevented perineal wound infection after APR [8]. Here, we report a case in which a combination of an omental flap with NPWT effectively avoided large pelvic dead space and prevented surgical site infection after TPE for anal fistula cancer.

## Case presentation

**Fig. 1:**
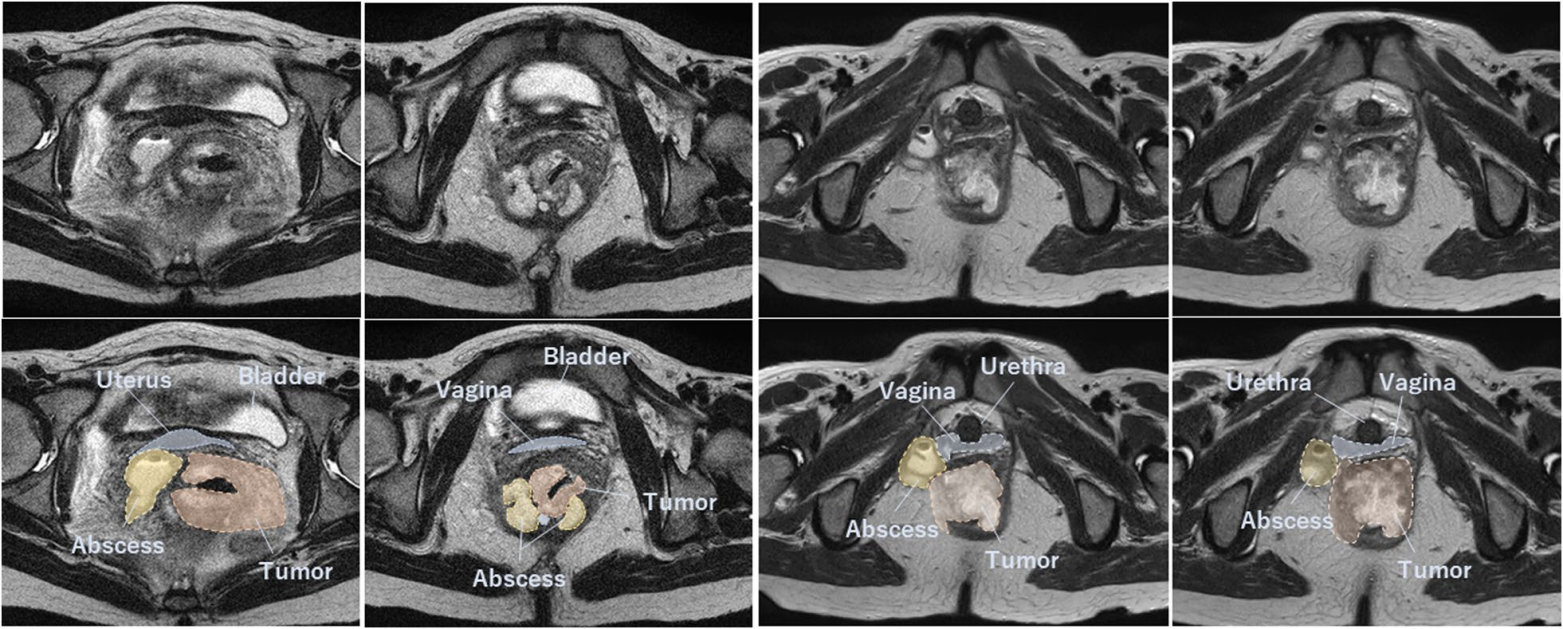
Preoperative axial pelvic magnetic resonance scans. Rb-p rectal tumor with abscess extending extensively into the pelvis and to the right side of the vagina and urethra. Blue area, uterus and vagina. Orange area, tumor. Yellow area, abscess

A 47-year-old woman with BMI of 15 had been diagnosed with Crohn’s disease involving the small and large intestine at 20 years of age. Although she had been treated with anti-tumor necrosis factor-alpha agents and was maintained in clinical remission, she had occasional recurrences of anal fistula. Sigmoidoscopy was performed to investigate anal pain and melena and showed anal stenosis and ulcerative lesions. Biopsy confirmed mucinous carcinoma. Preoperative pelvic magnetic resonance imaging revealed a perianal abscess arising from an anal fistula close to the tumor. The tumor was in contact with the right side of the vagina and the internal obturator muscle and extended to the vicinity of the urethra (Fig. 1). Biopsy of the anterior wall of the vagina revealed adenocarcinoma, which was suspected to be an extension of the tumor. The patient was diagnosed with anal fistula cancer with invasion to the vagina and the right side of the urethra. According to the 9th edition of the Japanese Society for Cancer of the Colon and Rectum guidelines [9], the tumor was clinically staged as cAI, cN1a, cH0, cP0, cM0, cStage IIIc. In the background that positive resection margin was a prognostic factor in anal fistula cancer, informed consent was given to the patient in consideration of these preoperative findings, and total TPE was selected [10]. The patient underwent staging laparoscopy and Seton drainage for infection and pain control. After the inflammation improved with drainage, TPE was performed via a combined laparoscopic and transperineal endoscopic approach [11].

**Fig. 2:**
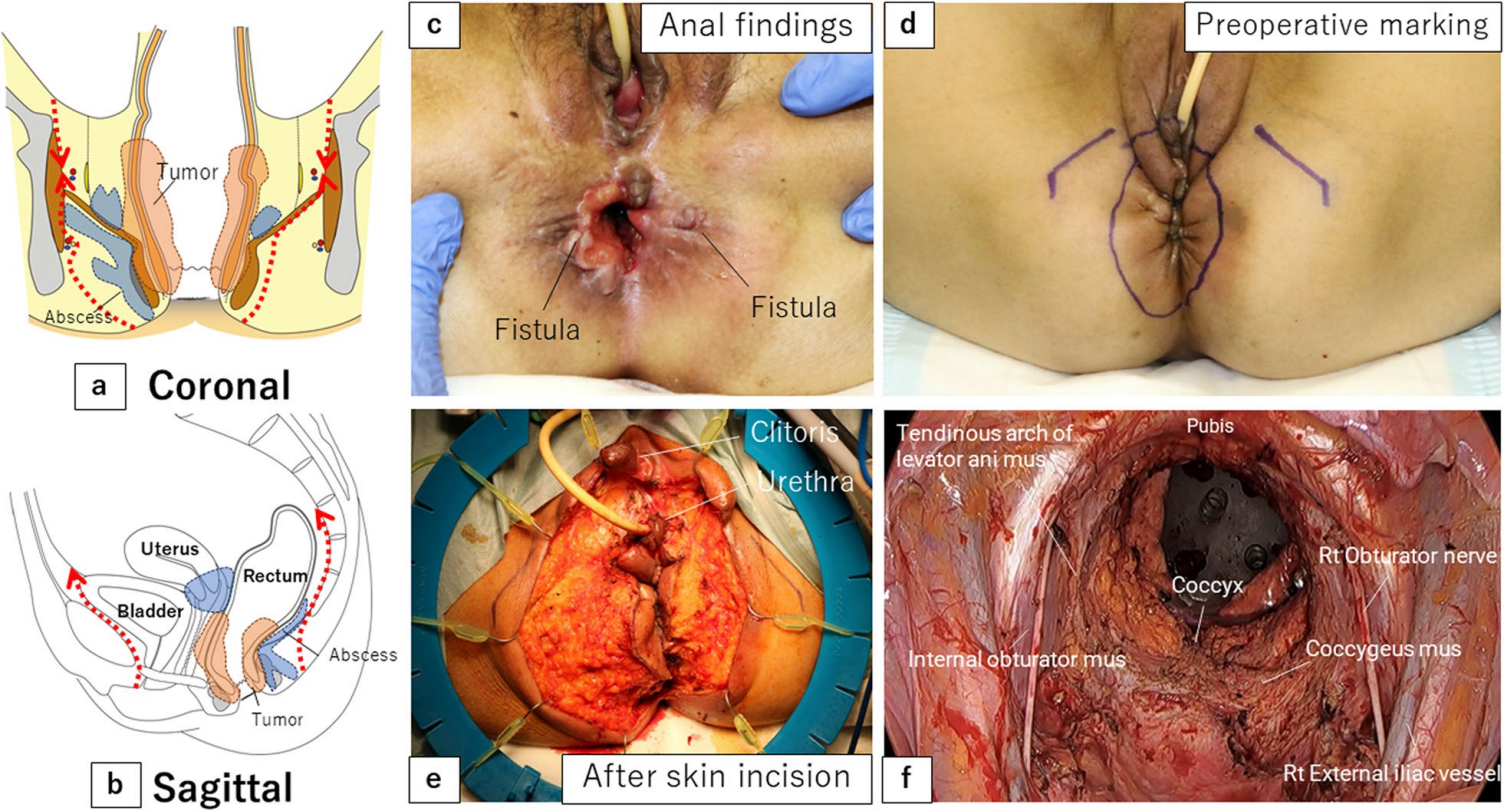
Preoperative and intraoperative findings. **a**, **b** Schema of the resection area. The abscess extends to the right side, and the perineum was extensively resected on the right side. Orange area, tumor. Blue area, abscess. Red line, resection line. **c** Preoperative anal findings: fistulas are present at 3 and 9 o'clock. **d** Preoperative marking of the resection line. **e** After skin incision. **f** After removal of a specimen

**Fig. 3:**
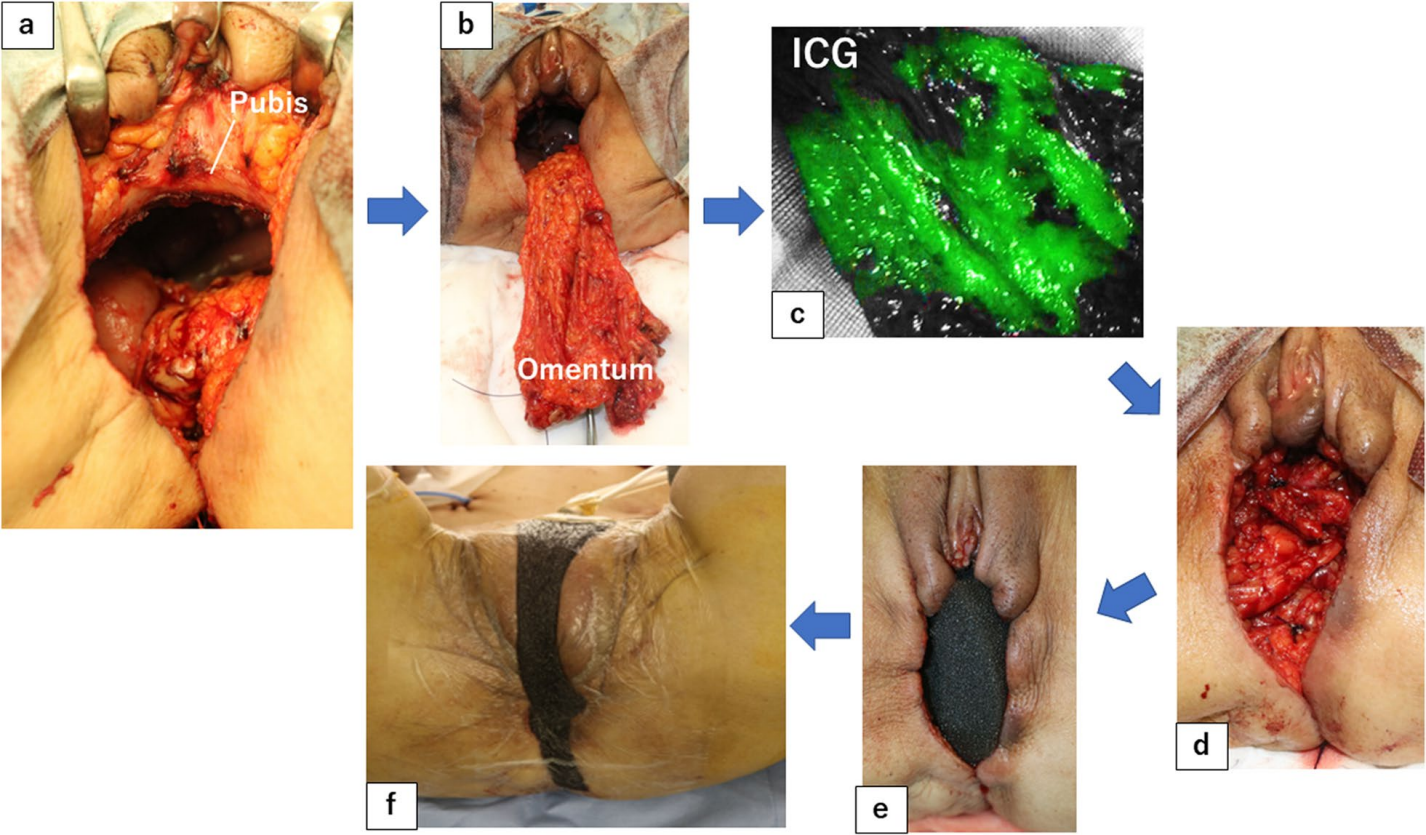
Methods for creating the omental flap and performing NPWTi-d. After ensuring the omentum was of sufficient volume and had adequate blood flow, it was mobilized to the perineal dead space, where the omental flap was created and NPWTi-d was performed. **a** Perineal findings after specimen removal. **b** Omentum after mobilization. **c** Adequate blood flow in the omental flap confirmed by intravenous injection of indocyanine green dye. **d** After application of the omental flap. **e** After application of the foam. **f** After application of negative pressure wound therapy with instillation and dwelling

**Fig. 4:**
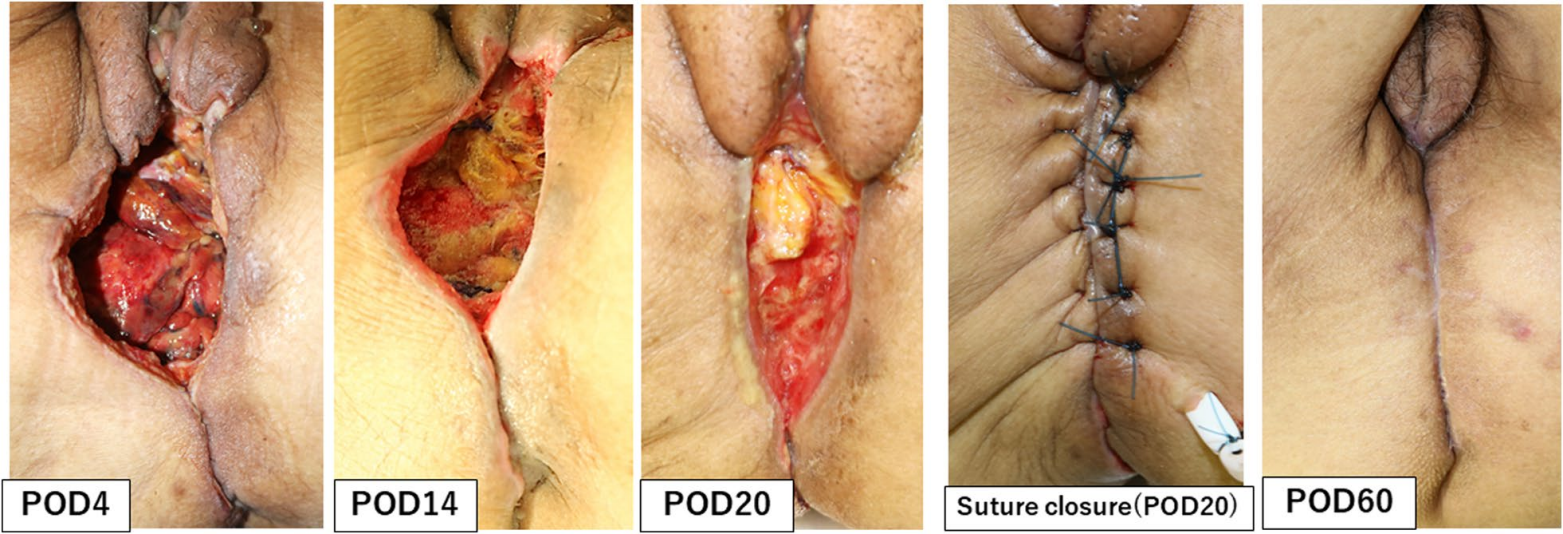
Healing of the perineal wound. Granulation tissue formed gradually in the pelvic dead space. The skin was closed on postoperative day 20

**Fig. 5:**
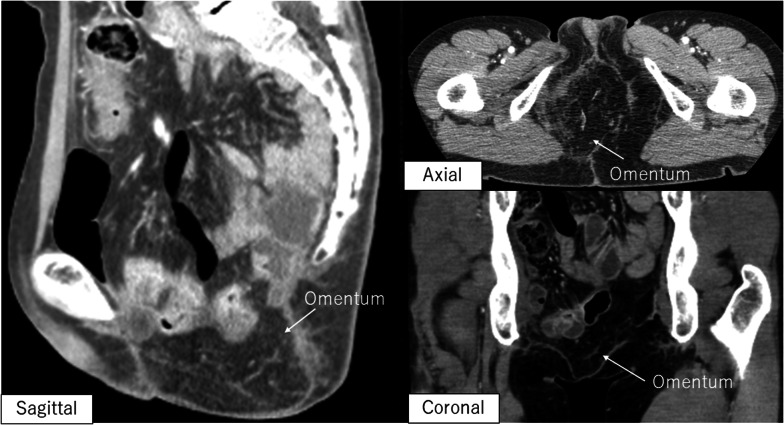
Postoperative computed tomography scan. The dead space in the ischiorectal fossa is filled with omentum. No perineal hernia is evident

After the vagina and anus including the fistula were closed with a purse-string suture (Fig. 2c, d), the skin was then incised to include the urethral opening and the external openings of the fistula on the excision side (Fig. 2e). On the right side, since there was an abscess in contact with the obturator internus muscle, the adipose tissue of the ischioanal fossa was extensively resected and the obturator internus muscle was partially resected (Fig. 2a, b). TPE was then performed using a combined laparoscopic and transperineal endoscopic approach. A ureterocutaneous fistula was fashioned for urinary tract reconstruction. The perineal wound measured 90 × 60 mm and primary skin closure was difficult (Fig. 3a). The volume of the omentum was considered sufficient to fill the pelvic dead space, so an omental flap and NPWT with instillation and dwelling (NPWTi-d) were used instead of a myocutaneous flap. The omentum was partially separated from the transverse colon, and the epiploic branch of the right gastroepiploic artery was sacrificed. The omentum was sufficiently mobilized to reach the perineal side (Fig. 3b). Injection of indocyanine green dye confirmed adequate blood flow to the omentum (Fig. 3c). The omentum was folded over several times and fixed to the perineal wound (Fig. 3d). The skin was closed with purse-string sutures and polyurethane foam for NPWTi-d was applied (Fig. 3e, f). The NPWTi-d protocol involved applying negative pressure with intermittent aspiration at − 75 mmHg until postoperative day (POD) 4 to prevent bleeding from the omentum, followed by aspiration at -125 mmHg. The foam was changed twice a week for 20 days after surgery. Granulation tissue in the perineal wound gradually formed (Fig. 4), and the wound was closed by suturing with a subcutaneous drain on POD20. A subcutaneous drain was removed on POD27. The patient was discharged on POD30 with no postoperative complications such as inflammation of the pelvic dead space, perineal wound infection, or ileus. At 9 months after surgery, she has no complications, such as perineal hernia, and there has been no recurrence of the anal fistula cancer. Computed tomography scans obtained postoperatively indicate that the omentum filled the space in the pelvis (Fig. 5).

## Discussion

TPE for anal fistula cancer is often performed with a myocutaneous flap to prevent infection in the pelvic dead space and the perineal wound [12]. However, a myocutaneous flap is invasive and reduces the patient’s quality of life. Charles et al. reported that use of an omental flap for large perineal defects after APR and TPE could decrease the incidence of postoperative complications [7]. An omental flap is less invasive than a myocutaneous flap and prevents loss of quality of life. Our patient underwent TPE for an anal fistula cancer with extensive abscesses, resulting in an extensive skin defect and a large pelvic dead space with the potential for bacterial contamination. Therefore, we used an omental flap to fill the pelvic dead space. An omental flap is preferable to a myocutaneous flap in a patient at high risk of pelvic infection because it can absorb tissue exudate in the pelvis, such as serum, lymph, and blood, and contains a rich vascular network that promotes migration of leukocytes to combat bacteria [13]. An omental flap is also easier to create and less invasive than a myocutaneous flap. The one disadvantage of the omental flap is that it cannot be used when mobilization is limited or the volume of the omentum is insufficient. Although they can be predicted preoperatively by CT scan, body size, and previous surgical history, it is not easy. If the intraoperative omental flap is difficult, a myocutaneous flap should be considered.

We also used NPWTi-d to prevent perineal wound infection and to promote granulation tissue formation in the skin defect. The omental flap also acted as a spacer between the small intestine and the polyurethane foam used for NPWTi-d, which allowed NPWTi-d to be performed more safely. In recent years, there have been some reports on the ability of NPWT to prevent perineal wound infection after APR [8]. NPWT accelerates wound healing by promoting angiogenesis and granulation [14–16], and NPWTi-d can increase removal of infectious exudate by perfusing saline into the surgical dead space.

There have been no previous reports on the use of NPWT in combination with an omental flap in cases of extensive skin defects and large dead space, as occurs post-TPE. The combination of an omental flap with NPWT can be useful for widely spread anal fistula cancer because it can help mediate the high risk of post-surgical pelvic infection and avoid the need for a myocutaneous flap. An omental flap could be a good choice when a sufficient volume of omentum can be mobilized into the pelvic dead space. However, more cases need to be accumulated to confirm the value of this method.

## Conclusion

We have reported a case of TPE for anal fistula cancer where we achieved a good short-term outcome by using an omental flap and applying NPWTi-d to prevent surgical site infection.

## Data Availability

Not applicable.

## Abbreviations

APR: Abdominoperineal resection
NPWT: Negative pressure wound therapy
NPWTi-d: Negative pressure wound therapy with instillation and dwelling
TPE: Total pelvic exenteration
